# Dental Pathology

**Published:** 1876-12

**Authors:** T. C. Brockett


### DENTAL PATHOLOGY.

BY DR. T. C. BROCKETT.

*Read before the Maryland State Dental Society.*

The president of this association having appointed me, one of its youngest members, to prepare a paper upon "Dental Pathology," it becomes me as an earnest working member to comply; but in the outset let me request that should my paper exhibit feebleness and disease in itself, it will receive a gentle treatment from those of my friends who have had more experience in the administration of therapeutical remedies; indeed if they are truly friendly, they will not fail to remember the established fact in medicine, that an enfeebled child can not survive the administration of strong medicine, but on the other hand small doses impart new life and vigor, until in time the system may almost become incapable of receiving a shock. I ask your attention here, briefly, then to a consideration of the "Diseases of the Dental Pulp;" and first, I may promise that such consideration must with every "dental practitioner" be a most serious one, as he is called upon more frequently to deal with such diseases than any other.

Under this head we may treat of the several troubles to which the "dental pulp" is liable, and as it is the particular province of the dentist to diagnose such cases, it becomes, therefore, a matter of importance that he be thoroughly posted in anatomy and physiology; indeed, no attempt to estimate the extent of the disease in an organ of whose normal state he knows absolutely little or nothing, would be barefaced quackery itself. As I have said, the importance of a knowledge of anatomy and physiology can not be overestimated. It would be just as impossible for a man to become a physiologist, without a knowledge of anatomy, as it would to become a pathologist without a knowledge of physiology. A thorough knowledge of the two is therefore among the essential requisites that make a good practitioner. The classification of the diseases of the "dental pulp" I have chosen from Salter's work on "Dental Pathology," recognized as the latest authority:

1st. Intrinsic Calcification.
2d. Suppuration and Sphacelus.
3d. Necrosis.
4th. Polypus.
5th. Sensitive Sprouting.

CALCIFICATION.

This condition is regarded as a low degree of morbidness, and is the result of some local injury or irritation, as but trifling causes are required to produce injurious effects upon the sensitive pulp of the tooth. Any injury to the external portion of a tooth will cause morbid nutritive activity within. Many teeth, which to a casual or superficial examination appear in a perfectly healthy condition have been previously the seat of disease. When calcification begins, there is first an impregnation of the tissues with a calcareous matter. These deposits are made at various points, and grow in proportion to the deposit, until by degrees the original centers come in immediate contact and form a solid mass, which, under certain favorable circumstances, forms "Osteo-Dentine." When calcification is complete, the whole mass becomes as hard as ivory; but at any time previous to the completion of the process of calcification, the mass may be disintegrated and torn into fibrous shreds. Solidification takes place first in the axis of the pulp, this part being complete, while the remaining portion, the exterior, continues rather soft, until the "Osteo-Dentine" becomes fused and confounded with the dentine.

As a rule, the more decayed the tooth, the more calcified will be the pulp.

In the process of calcification, so far as my observation goes, there is no discoloration of the tooth substance, but the walls of the pulp cavity are as white and clean as though no change had taken place, showing how beautifully nature performs her work.

### SUPPURATION AND SPHACELUS.

This is one of the commonest troubles to which the pulp is liable, and is often frequently the result of bad practice on the part of the dentist. I regret to say that there are many men who think far more of their fee than they do of preserv- the teeth which are intrusted to their care. I have frequently had cases come under my immediate observation, where men have filled the main cavity of a tooth and left the dead pulp. This, as you are aware, always results in disease if not in the total destruction and loss of the tooth; and when a patient returns to his dentist and asks the reason of such a result, what is his answer? It is invaribly, you have taken cold in it. This, gentlemen, is the way some men have of shielding themselves and disgracing their profession. This trouble, like others, is capable of a variation of intensity. Sometimes, for instance, we may have simply the formation of a globlue of pus, being the result of some slight inflammatory action. And at other times we may have complete suppuration and sphacelus of the organ. This is a much more serious form of the disease, and generally results in alveolar abscess.

Salter holds "that we may have alveolar abscess without suppuration of the pulp. And, on the other hand, that the pulp may suppurate without the discharge finding an exit." The phenomenon of tooth suppuration is identical with suppuration of any other organ. But as a rule we have more pain attending suppuration in a tooth than elsewhere. The reason of which is obvious:

First. The pulp of a tooth being so abundantly supplied with nerves, renders it exquisitely sensitive. And again, to the manner in which the pulp is encased. The bony chamber in which it is enclosed is of such a firm and unyielding nature as to render expansion impossible; and in this way, when the suppurative process sets up and the pus is exuded and poured into the pulp chamber, it causes a pressure upon nerves which is almost intolerable. In studying the pathology of the teeth we must remember that the teeth have two sources of vitality,—the periosteum as well as the pulp,—and while we may destroy the pulp, there is still remaining a certain amount of nourishment and vitality, which is derived from the peri-dental membrane, and, therefore, it is not until the total destruction of both that we may consider a tooth dead.

A very interesting case came under my observation in September, 1873. Mr. H., a well known merchant in this city, (Baltimore,) was attacked with a severe tooth ache, so severe indeed, that it unfitted him for business. The tooth was a right second superior molar, which had the nerve killed some three or four years previously. The tooth had been filled, but the dead nerve had not been removed. He says he had occasionally felt some soreness in the tooth, but it was not until the above named time that the tooth ached so as to demand its immediate removal. This was done, and upon an examination an abscess was discovered upon one of the fangs. Mr. H. returned to his work feeling sure that he had got rid of the offending organ, but to his surprise in about twenty-four hours thereafter his face commenced to swell, and he accordingly called upon his family physician, who prescribed a flax seed poultice. This treatment was continued for fourteen days, when the abscess pointed on the cheek just under the malar bone, at which time it was opened and a large amount of pus escaped. This continued for some months without relief, when at last Mr. H. determined to go to Philadelphia and remain there under treatment until cured, which he did about six months ago, and Dr. Pancoast operated upon him by cutting through into the antrum, the seat of the trouble, and treating it through this opening. Mr. H. improved so much in a few weeks that Dr. Pancoast advised him to return, assuring him that he would experience no more inconvenience, but before his departure, however, the doctor inserted a small silver tube in the opening, which can be removed at pleasure, thereby securing an exit for any pus which may form. The flow of pus has been so much diminished now that he expects entire relief in a few weeks.

### NECROSIS OF THE PULP.

This is generally the result of some mechanical injury. A blow or a fall will frequently sever the vessels at the foramen and thereby cause necrosis. Inflammation often terminates in necrosis.

Works upon pathology mention necrosis of the pulp as occurring spontaneously. My own belief upon the subject is that where we find this trouble, it is the result of one of the above named causes, and not spontaneous. An inflammatory action may take place in a tooth and result in necrosis of the pulp without giving rise to any very serious pain, and when the inflammation subsides the tooth may appear perfectly healthy. The same result may be had from a blow, causing little or no pain at the time, but terminating finally in the death of the pulp. About two years ago a gentleman called at my office to have his teeth filled. Upon an examination I found the left central decayed upon the anterior approximal surface. The decay was not extensive, and required but a simple filling. Having filled that tooth, together with several back teeth, the patient was dismissed. Before leaving the office I told him that he should have his teeth examined about twice a year, which he promised to do, but as he was not a resident of the city I saw nothing more of him for about twelve months, when he called with his face very much swollen, and informed me that the front tooth which I filled for him was causing him intense pain, and had been for several days. I must admit I was greatly astonished at such a revelation, but I was not prepared to believe it, for I knew that I had not filled over an exposed nerve and thereby caused necrosis by an inflammatory process, nor had I been guilty of filling a dead tooth without opening the nerve chamber. But an examination resulted in a diagnosis which relieved me of all my personal responsibility, as the tooth which I had filled was not the diseased one, but it was the right central. This tooth, though without a decayed spot upon it, was in a most horrible condition. It was discolored, loose, and from around the neck just under the margin of the gum pus was exuding. Finding the pus had an outlet, and he face was so much swollen that I could not operate, I determined to wait for a few days, at which time I opened the pulp chamber from the palatine surface, so as to reach the fang by a direct line, and treated the tooth and filled it with a very happy result. Previous to operating I questioned my patient concerning it. He first denied ever having received a blow or a fall, thereby subjecting it to mechanical violence, but suddenly he remembered that about three years previously he was kicked in the mouth by a gun shot by himself, the result of which was the loosening of this tooth, but he said that after a week or two it became firm again, and he had never thought about it since. This serves to demonstrate a case of necrosis of the pulp resulting from mechanical injury. I shall now briefly refer to—

POLYPUS OF THE PULP.

This a trouble which is less frequent than those of which I have just spoken. It usually commences by the exposure of the pulp in a tooth which has not been perfectly calcified and which has never ached. The second set of teeth are much more liable to this disease than the first, although the first set does not entirely escape.

We find polypus oftener in the teeth of young than in older persons, and it is not an unfrequent occurrence to find one or more teeth affected in the same mouth. The best treatment is to remove the tooth, as the decay has usually progressed to such an extent as to demand their removal; and, besides, there is little or no certainty resulting from extirpation with the knife.

SENSITIVE SPROUTING OF THE PULP

is fortunately a form of tooth trouble which we do not very frequently meet with, but when we do, it is attended with great pain to the patient. The tooth is exquisitively sensitive to the touch, as well as to either heat or cold.

The best treatment is to remove the tooth, as the growth is very apt to return when extirpation is resorted to. This condition is the result of mechanical violence, caused sometimes by the fracture of a tooth in an effort to extract it, but more frequently by a fall or a blow. Upon the exposure of the pulp, a growth or sprout takes place, and sometimes attaining the size of a raspberry and resembling it in the nodules which it presents upon its surface. Upon an examination with the microscope, this sprouting resembles the insensitive polypus, but presents a clearer and softer appearance, together with a more abundant supply of nerves. Cases have been mentioned where cures have been brought about by the parts sloughing away spontaneously. Mr. Salter relates a case of sensitive sprouting which he cured by accident. A medical student from "Guys Hospital" applied to him (Nov. 9th, 1864) on account of a fractured first upper molar, which had been broken off in an attempt to extract it. The large pulp of the tooth's central chamber was exposed and intensely sensitive. He attempted the extraction with an elevator, and lifted the tooth from the socket, when the patient plunged away declaring he would stand no more torture. The tooth returned to its alveolar cavities, but the pain was cured. In prying up the fangs, the connection of the pulp nerves with the maxillary nerve had been severed, and the case was so far cured, the tooth remained and the periosteal union was re-established. The pulp sloughed away.

In thus presenting by the limited means of an essay the diseases of the dental pulp, I am aware that the field is much more comprehensive than I have above laid down; yet if my imperfect reflections shall incite some member of our honorable profession into a more careful research, there is no one who will hail the discovery of new facts more than myself.

